# Contribution of Mucins towards the Physical Properties of the Tear Film: A Modern Update

**DOI:** 10.3390/ijms20246132

**Published:** 2019-12-05

**Authors:** Georgi As. Georgiev, Petar Eftimov, Norihiko Yokoi

**Affiliations:** 1 Department of Optics and Spectroscopy, Faculty of Physics, St. Kliment Ohridski University of Sofia, 5 James Bourchier Blvd, 1164 Sofia, Bulgaria; ggeorg@phys.uni-sofia.bg; 2 Department of Cytology, Histology and Embryology, Faculty of Biology, St. Kliment Ohridski University of Sofia, 8 Dragan Tzankov Blvd., 1164 Sofia, Bulgaria; peftimov@uni-sofia.bg; 3 Department of Ophthalmology, Kyoto Prefectural University of Medicine, Kyoto 602-0841, Japan

**Keywords:** tear film properties, secretory mucins, membrane associated mucins, dry eye therapy

## Abstract

Instability of the tear film (TF) protecting the ocular surface results in dry eye syndrome (DES), the most prevalent public health ophthalmic disease affecting the quality of life of 10 to 30% of the human population worldwide. Although the impact of the tear film lipid layer (TFLL) and of the aqueous tears (AT) to the TF stability is extensively studied, in contrast the contribution of the secretory mucins (SM) and of the membrane-associated mucins (MAM), i.e., one of the most abundant molecular classes in AT and in the corneal epithelium respectively, remains poorly defined. However, it is well known that in DES both types of mucins are quantitatively or qualitatively deficient. Numerous studies since the 1990s until now have proposed direct involvement of SM and MAM in the material properties (viscoelasticity, hydration, and protection of the ocular surface; synergistic cooperation with the rest of the TF layers; etc.) and stability of TF. These theories will be reviewed here in the context of the classical and modern in vitro and in vivo results that allow their reappraisal and in view of the novel mucin secretion enhancing pharmaceuticals, which have opened innovative routes for the therapy of DES.

## 1. Introduction

Tear film (TF) is composite wetting film which consists of tear film lipid layer (TFLL) at the air/tear surface and underlying aqueous tear (AT) positioned over the glycocalyx of the corneal epithelium [1,2]. When some of the TF layers and/or the glycocalyx of the corneal surface epithelium are quantitatively or qualitatively affected this results in TF instability leading to dry eye syndrome (DES). DES is the most prevalent public health ophthalmic disease affecting the quality of life of 10–30% of the human population worldwide [3]. It has gross socioeconomic impact in developed societies: the direct expenditures of treating a patient ($783 per year) and the burden of work productivity loss and societal impact ($11,302 per person yearly) sum up to $55.4 billion annual cost of DES for the US population [4] with the figures estimated to be similar across the globe [5].

Although the contribution of TFLL and AT volume to the TF stability is extensively studied, in contrast the contribution of the secretory mucins (SM) and of the membrane associated mucins (MAM), i.e., one of the most abundant molecular classes in AT and in the corneal surface epithelium respectively, remains poorly defined [1,2]. However, it is well known that in DES both types of mucins are quantitatively or qualitatively deficient (normally by changed ratio of O- and N-glycosylation or decreased glycosylation) [6]. It was speculated that SM and MAM alterations correlate with inflammation [7] or osmolarity [8], both of which however are not necessarily directly cause for shorter breakup time (BUT) of TF. The same is valid for the role of MAM as a barrier to the invasion of pathogens in the corneal epithelium [7]. Numerous theoretical studies since the 1990s until now has proposed far more direct involvement of SM and MAM in the material properties and stability of TF [9,10,11]. These theories will be reviewed here in the context of the classical and modern in vitro and in vivo results that allow reappraisal of their validity and clinical relevance.

## 2. Contributions of Secretory Mucins to the Stability of Human Tear Film

### 2.1. Shear Thinning Property of Whole Human Tears

**Figure 1:**
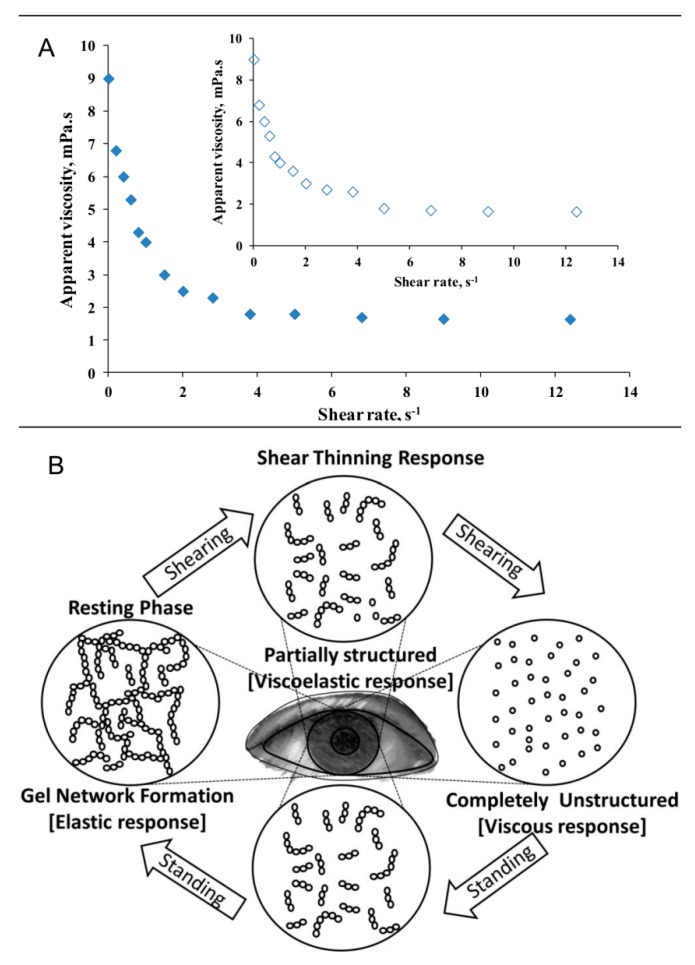
(**A**) Viscosity as a function of shear rate for stimulated (open dots; insert) and unstimulated (filled dots) human tears. (data from Tiffany 1998, [15]). (**B**) schematic presentation of the reorganization of shear thinning 3D structure like the AT at shear and in rest (adapted from [20]).

Shear thinning property of human tears, stimulated and unstimulated, is well known (Figure 1) and it is one of the most important properties of AT [12,13,14,15,16]. As can be seen at Figure 1 at shear rates ≥ 6 s^−1^ the AT is with water like viscosity (~1cP) and represents unstructured colloid solution; this means that at blink it serves as non-viscous lubricating fluid which prevents the dry friction between the back of the eyelid and the eye ball surface, and simultaneously the low AT viscosity avoids viscous drag over the delicate corneal epithelium during blink [10,11,12,13,14,15,16]. In contrast, when left at rest, AT compounds form ternary gel (viscosity as high as 9 cP) that resists meniscus induced thinning [17,18] and adds tensile strength to the TF [19]. The range of possible structural reorganizations of colloid solutions like AT at shear and at rest are schematically represented at the panel B of Figure 1.

Number of hydrodynamic models (see Equations (1) and (2)) were proposed which clearly emphasize that as higher is the viscosity (µ) of AT at rest in open eye as higher is the TF thinning time and non-invasive breakup time (NIBUT) value [17,18].

(1) $$t=13.6{\left(\frac{h_{0}}{R}\right)}^{1.2}{\left(\frac{R}{h_{m}}\right)}^{2.2}\frac{\mu R}{\sigma }$$

(2) $$t=3.52\;\left(\frac{R}{h_{m}}\right)2.232\left(\frac{h_{0}}{R}\right)1.268\left(µ\frac{RC}{\sigma }\right)$$

Here, *t* is TF thinning time, R is tear meniscus radius, h_o_ and h_m_ are the initial and minimal TF thickness respectively, µ is tear viscosity, and σ is tear surface tension. Equation (2) [17] also contains the term C which accounts for the capability of the TFLL to provide tangentially immobile air/tear surface (Gibbs–Marangoni effect).

Recently, it was clinically demonstrated that indeed the extensional viscosity of dry eye tears is compromised compared to healthy samples [21]. Back in 1991 Tiffany also demonstrated that DES tears take longer time to stabilize after deformation is applied to them, which also is indicative for less ordered and less viscous fluid in an open eye (i.e., in resting phase at the bottom panel of Figure 1) [12].

Although the importance of AT viscosity is well grounded theoretically and relevant clinically it turns out that the molecular origins of the shear thinning property are far less clear.

Secretory mucins like MUC5AC are well known to form shear thinning hydrogels in the human body (in the respiratory airways and in the gastrointestinal tract) and the hypothesis for their crucial role for this AT property has found its place in multiple publications [6,7]. At the same time, the quantitative experiments on the topic performed in the classical studies by Tiffany in the period 1990–2005 reveal far less clear picture. Based on densitometry measurements of spots in electrophoretic gel with rabbit ocular mucin used as control he estimated the secretory mucin concentration in capillary collected human tears to be up to 0.125 mg/mL [15]. Subsequent immunoassay estimations of Schirmer strips extracts suggested that the MUC5AC concentration in ‘healthy’ tears was ≤ 200 µg/mL porcine stomach mucin equivalent [22] and 232.3 ± 22.3 μg/mL [23]. At such concentration commercial submaxillary mucin (the commonly preferred mimic of MUC5AC) is too diluted and was not able to display shear thinning properties alone or in combination with other tear constituents [13,15,16]. Interestingly, delipidation of human tears also resulted in loss of shear thinning property although lipid-loaded holo-lipocalin (thought to be the major carrier of lipids in AT) was not able to show non-Newtonian behavior [15,16]. In contrast, any binary or tertiary model mixture of tear proteins that contained physiological amounts of lysozyme or lactoferrin showed shear-thinning property. Tiffany concluded that the non-Newtonian behavior of whole human tears “cannot be explained by the amount of mucin present” and that “hetero-protein interactions, possibly electrostatic, involving lipid-binding-induced structural changes to tear lipocalin, significantly contribute to the viscosity of human tears” [16].

The reasons for such discrepancy between the hypothesized role of the gel-forming MUC5AC for the shear thinning-properties and the underlying structure of tears in open eye and the experimental findings of Tiffany might be due to couple of reasons. Firstly, it was recently shown that the method of collection of tear samples (microcapillary, Schirmer strips, etc.) may result in very different amount of mucins, lipids, and other compounds in the specimen which considering the complex hetero-molecular interactions behind the non-Newtonian behavior of human tears may grossly impact the sample’s performance and composition [24,25]. Secondly, the commercial mucin preparations used in the experiments of Tiffany (primarily bovine submaxillary mucin) have very different levels of N- and O-glycosylation compared to ocular mucins [6,7] which certainly will affect their hydrogen bonding pattern and rheological properties.

Furthermore, there is significant amount of clinical evidences suggesting the crucial role of tear secretory mucins for the gel like structure of tears in open eye during the interblink. These will be discussed in detail in the next point.

### 2.2. Aqueous Tear Gel as a Surface Chemical Protection of the Ocular Surface

Apart from providing mechanical stability to the TF in open eye, the muco-aqueous gel (MAG)—i.e., the shear thinning gel-like structure formed in the AT bulk (Figure 1)—was demonstrated to act both as surface-chemical trap for the capture and removal of hydrophobic contaminants (lipids, dust particles, cell debris, etc.), and also as a surface-chemical barrier preventing the epithelial contamination [11]. It is now well known that due to the presence of membrane associated MUC 16 and other glycocalyx components the surface of corneal epithelium shows excellent wettability by itself [6,7]. Still if it gets deprived of MAG coverage the attachment and accumulation of the contaminants on the epithelium becomes energetically possible, which can mask the hydrophilic nature of the cell glycocalyx [11].

As secretory gel-forming mucins like MUC5AC are well known to perform such protective functions in the lining of the alveoli and of the gastrointestinal tract that role was readily ascribed to MUC5AC (being the most abundant secretory mucin in human tears) at the ocular surface [6,7]. Although such hypothesis does not correlate well with the previously discussed laboratory studies of Tiffany, the in vivo and clinical data look more supportive for the key role of MUC5AC (in interplay with other tear constituents, i.e., proteins and lipids) for the formation of mucoaqueous protective and shear thinning gel. These in vivo data are briefly summarized below.

The term mucin-deficient dry eye was first coined by Lemp in the 1970s [26,27,28,29]. He demonstrated that in the absence of mucus-secreting goblet cells, the tear film of patients with normal AT volume and no signs of meibomian gland dysfunction (MGD) ruptures in about 3–5 s following a blink [26]. Subsequently multiple evidences were found in biochemistry studies that the amount of MUC5AC decreases up to three times in dry eye patients and the degree of O- and N-glycosylation is also altered in DES sufferers compared to healthy individuals [6,7,30,31,32]. However, as in most of these studies the MUC5AC alterations were accompanied by changes in other TF compounds the implication of MUC5AC to TF stability was not straightforward. A more unambiguous interpretation can be made based on studies in which the tear secretory mucins, MUC5AC in particular, were specifically targeted.

First such clues were provided by animal models of dry eye that employ chronic exposure to the anti-cholinergic agent scopolamine to reduce goblet cell secretion of MUC5AC [33,34]. These studies revealed that Lewis rats developed TF instability within two days of scopolamine treatment. The studies by Floyd et al., 2012 revealed that MUC5AC knockout mouse model had the same AT volume compared to wild type animals, but their TF was unstable (4.3 s in KO mouse vs. 10 s in wild type animals) [35]. Furthermore, a 10× increase in corneal fluorescein staining was observed in KO mouse, which strongly supports the hypothesis for the role of MAG to the defense of the corneal epithelium integrity. Other experiments with MUC5AC and MUC5B null mouse [36] did not reproduced these findings probably because of the multiple differences in the experimental protocol: difference in vivarium environments for the mice; alterations in the microscopy protocol for assessment of goblet cell morphology (paraffin versus methacrylate preservation); different Muc5b antibodies. However, the results of Floyd et al., 2012 [35] strongly correlate with recent clinical findings on the impact of MUC5AC. It was shown that MGD in stearoyl-CoA desaturase-1 KO mice is associated with compensatory increases in tear volume and mucin levels, which stabilize the TF [37]. Furthermore, the instillation of 3% diquafosol ophthalmic solution had immediate (within 15 min of instillation) TF stabilizing effect in keratoconjunctivitis sicca rat models which precisely matched the time course of the drug induced increase in the secretion of MUC5AC in tears [38]. Thus, although many points need further study the bulk amount of in vivo data strongly supports the importance of secretory mucins, especially MUC5AC, for the colloid structure of AT in open eye and for its contribution to the overall stability of the tear film.

### 2.3. Secretory Mucins as Spreading Agents for TFLL

**Figure 2:**
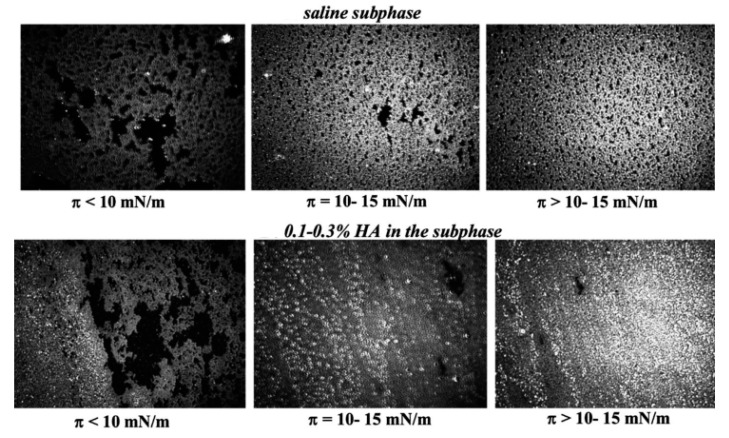
Brewster angle microscopy (BAM) micrographs (500 × 500 µm) of human meibum films obtained in Langmuir trough representative of the designated surface pressure (π) range. It can be seen that at π ≥ 10 mN/m the mucomimetic polymer hyaluronic acid (HA) results in more uniform and thicker (i.e., brighter) meibomian layers (with permission, from Georgiev et al., 2013) [40].

It was recently found in number of in vitro studies (see Figure 2) that the uniform spreading of meibomian and tear lipid films at the air/water surface is enhanced by polymers like the ones found in AT that contain polyanioic polysaccharide moieties, e.g., hyaluronic acid and secretory mucin glycoproteins [39,40,41,42].

It can be attributed to the formation of polymer interfacial gel-like networks due to H-bonding of the polymer moieties, each with other and with the polar lipids headgroups, thus resulting in increased film viscosity, more uniform 2D distribution of the lipids and water incorporation in the films [2,40]. This is a rapid mechanism, on the physiologically relevant time scale of seconds, which demonstrates that all the layers of the tear film should be viewed not as separate entities but as the parts of a continuous system in constant interplay between each other.

The importance of mucin-like polymers for the spreading of the TFLL were confirmed clinically by the capability of 0.1% HA for 15 min post-instillation to increase the thickness of TFLL of lipid deficient patients from <60 nm to 75 nm [43] and by the similar impact of 3% diquafosol ophthalmic solution (a drug promoting the rapid secretion of MUC5AC) on the TFLL in healthy human eyes for more than 1 h post-instillation [44].

## 3. Contributions of Membrane Associated Mucins to the Stability of Human Tear Film

### 3.1. MAM and Corneal Wettability

In order for TF to be stable, it is necessary for the corneal surface to be fully wettable, otherwise instantaneous TF breakup will take place at the region where dewetting occurs [11]. The wettability of the corneal epithelium surface is determined by the intact structure of the glycocalyx and membrane associated mucins (MAM) [11,14,45]. It is well known [45] that “MUC4 and -16 are particularly important hydrating molecules of the ocular surface due to the drying seen on the ocular surface with vitamin A deficiency” (vitamin A is essential for the stimulation of the expression of both MAM). The lack of sufficient vitamin A causes abnormal differentiation of the ocular surface epithelium, resulting in keratinization of both conjunctival and corneal epithelial cells, termed xerophthalmia [46].

**Figure 3:**
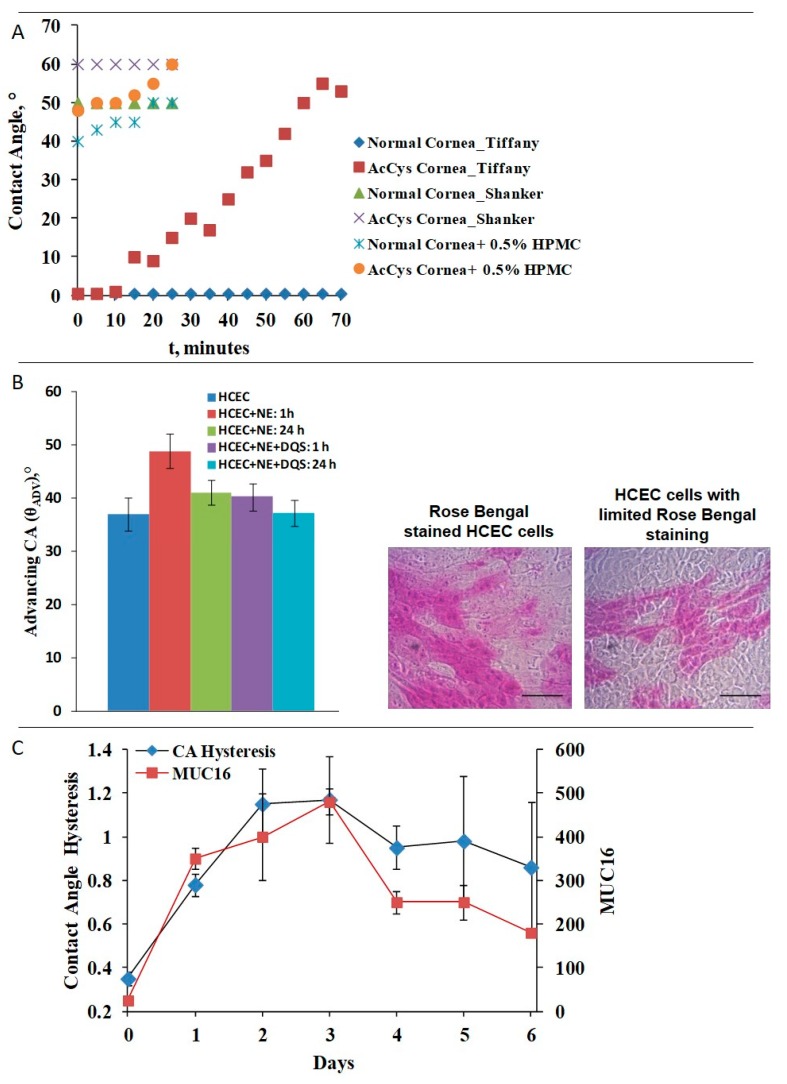
Ocular surface wettability data. Panel (**A**): contact angles of enucleated rabbit corneas (data from Tiffany, 1990 [12]; Shanker et al., 1995 [45]). Panel (**B**): contact angles of stratified cultures from HCEC (human corneal epithelium) cells; bar = 100µm (with permission from Georgiev et al., 2015) [48]. Panel (**C**): contact angle hysteresis and MUC-16 expression of stratified cultures of hTCEpi cells (data from Yáñez-Soto et al., 2015) [49].

Here very valuable quantitative research was pioneered once again by Tiffany [14]. He measured the changes in the water contact angle of 0.5 µL water drops positioned on multiple positions on freshly enucleated rabbit cornea left to dry in air. The contact angle remained 0° (i.e., ideal wettability) for 90 min after cornea is enucleated and left to dry in air (Figure 3, panel A). In contrast, enucleated rabbit corneas briefly rinsed with acetylcysteine showed rapid increase of contact angle to 10° that gradually rose further. N-acetylcysteine (NAC) is known to affect the inter- and intramolecular disulfide bridging capability of the SEA (sea urchin, enterokinase and agrin) modules of the MUC16 molecules, i.e., in order to deteriorate corneal wettability it is not necessary to shed MAM but merely to impair its extracellular domain. Similar results on water contact angles were demonstrated with enucleated rabbit corneas by Sharma et al., 1998 with sessile drop measurements, and ex vivo by Shanker et al. 1995 (Figure 3, panel A) who utilized captive bubble setup to access the contact angle of enucleated rabbit cornea kept hydrated in a measurement chamber [11,47]. The somewhat higher contact angles obtained by Shanker et al. and by others [47,48,49] compared to the values reported by Tiffany [14] can be attributed to the difference in the contact angle measurement methodologies. While Tiffany [14] used sessile drop technique, rest of the studies [47,48,49] were performed with captive bubble setup. In the latter case, the direct contact of the air bubble with the underlying corneal epithelium, as well as the pressure exerted by the bubble in the contact zone can result in slight shear of the MAM ectodomains and in partial exposure of the less hydrophilic cell membranes beneath the MAM enriched glycocalyx [11]. In the study of Shanker et al. [47] it was also demonstrated that in conditions resembling the AT turnover by application of water flow in the measurement chamber mucomimetic polymers like hydroxypropyl methylcellulose (HPMC) adhered stronger and more durably to corneas with intact corneal epithelium than to NAC treated one. The MAM 16 shedding of stratified culture of human corneal epithelium (HCEC) cells (Figure 3, panel B) with neutrophil elastase resulted in 15° increase of the contact angle which recovered very slowly in time [48]. The supplementation of the cells with 3% diquafosol sodium (a MUC16 secretatogogue) resulted in rapid recovery of MUC16 (followed by the resistance of HCEC cells to rose Bengal staining) within 1 h of instillation and almost complete recovery of corneal cells contact angle after 24 h. The importance of MUC16 to the wettability of cell cultures was also demonstrated in experiments with stratified immortalized human corneal epithelial cell line (hTCEpi) cultures [49] where the time course of MAM16 expression correlated with the surface chemical heterogeneity and with the contact angle hysteresis of the cell cultures (Figure 3, panel C). Multiple studies in immortalized human corneal-limbal epithelial (HCLE) cells [50] have found that MUC16 is responsible for the antiadhesive property of the corneal surface, which is also a manifestation of highly wettable and hydrophilic surface.

The in vivo animal model and clinical results also strongly suggest the importance of MUC16 for the wettability of the corneal surface and for the stability of TF. It is shown that the distribution of MUC16 loses its uniformity in dry eyes as compared to healthy ones [51,52] and greatly differs between mature and undifferentiated corneal epithelium cell cultures [49].

Furthermore, recently animal models for MAM deficiency were designed by topical application of 10% NAC in male Sprague-Dawley rats for 5 days, four times a day [53]. NAC treated rats showed significant (*p* < 0.01 compared to controls) decrease in tear secretion, corneal wettability, BUT of TF, tear MUC5AC concentration, and numbers of conjunctival goblet cell. In addition, significant raise in corneal fluorescein score and Rose Bengal scores were observed in the NAC group (*p* < 0.05 and *p* < 0.01, compared to controls respectively).

Compromised corneal wettability looks the only plausible explanation for the short BUT (SB) dry eye characterized with instantaneous spot-like fluorescein breakup (FBUT = 0 s) immediately at eye opening, i.e., at the AT deposition stage prior TFLL spreading to take place [54]. As in short BUT, TFLL properties and AT volume are normal, locally impaired wettability of the corneal surface at the breakup spot region appears as a probable cause for the TF instability. Such possibility agrees with the theoretical predictions that the TF breakup can be initiated even by a very small, mildly non-wettable corneal site with the size of a single superficial squamous cell (i.e., ~20–40 µm diameter) [11]. The deficiency or the impairment of MUC16 is thought to be the most probable reason such a discrete mildly non-wettable corneal site to appear [55,56,57]. Another possibility is that, the glycocalyx becomes contaminated with lipids, e.g., because of a dimple formed below lipid ‘globs’ (lipid particles supposed to exist at the AT surface prior the spreading of the major part of the TFLL) [58]. Both mechanisms might also act synergistically. It is interesting to note that diquafosol (P2Y2 purinergic receptor agonist) appears to be highly efficient treatment of short BUT dry eye. The instillation of diquafosol sodium eye drops reportedly rapidly (i.e., within 15-min post instillation) increased the volume of aqueous tears in healthy [59] or ‘dry’ human eyes [60], the secretory mucin content in normal human eyes [61], and the gene expression for membrane-associated mucins (MUC1, 4, and 16) in cultured human corneal epithelial cells [62].

**Figure 4:**
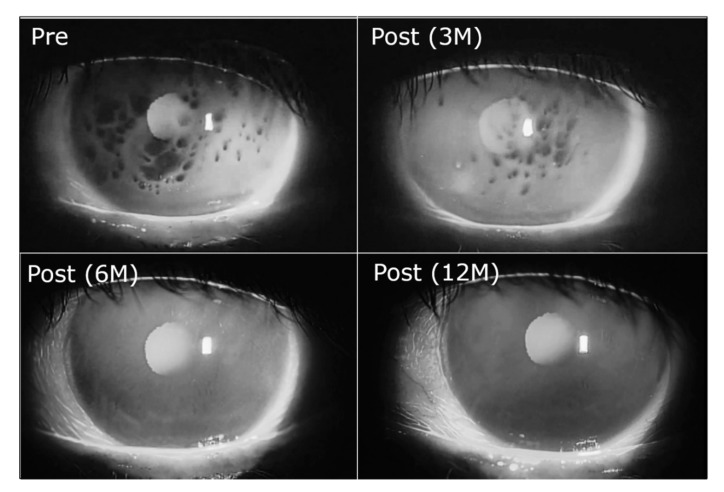
Dry eye with spot break treated with 3% diquafosol sodium eye drops (6 times daily). Spot break disappeared 6 months after the treatment and the disappearance is maintained even in 12 months after the treatment (Pre: before the treatment, Post: after the treatment, M: months) (with permission from [63]).

It was found clinically that after months of treatment with 3% diquafosol sodium eye drops gradually the SB pattern recovers to normal [60,63] (Figure 4), thus closely matching the anticipated course of its MUC16 recovering action [62,64]. In short, BUT dry eye the breakup areas are of the ‘dots’ type (small regions not expanding after breakup), which also suggests discrete local impairment of the hydrophilic corneal epithelium glycocalyx. Normal glycocalyx covered with MAG layer is supposed to be resistant to short-term contact with lipids, which results in mobile and transient thinning below the lipid ‘glob’ without true pinning to the corneal surface to occur [58].

### 3.2. MAMs and the Ocular Surface Lubrication

Microtribometry measurements mimicking the conditions at the ocular surface show that healthy corneal surfaces have extremely low coefficient of friction when bathed in physiologically relevant lubricating solutions. Experiments with 28 fresh human donor corneas with intact epithelia revealed that mean (SD) CoF values ranged from 0.006 to 0.015 and were 0.013 (0.010) in TMS-PS (tear-mimicking solution in borate-buffered saline), 0.006 (0.003) in TMS-PBS (TMS in phosphate-buffered saline), 0.014 (0.005) in TMS-HEPES (TMS with HEPES-buffered saline), and 0.015 (0.009) in TLF-PBS (tear-like fluid in PBS) [65]. Study with stratified cultures of mucin-producing human corneal epithelial cells (hTCEpi) showed μ = 0.058 ± 0.008 [66].

Such extreme lubricity and very low CoF is attributed to the boundary lubrication action of MUC16 and rest of the MAMs involved in the formation of the glycocalyx coupled with the lubricating properties of the MUC5AC-enriched AT preventing the direct ‘dry’ contact between the eyelid wiper and the corneal surface [67,68,69,70].

Although no in vitro measurements are available on MUC16-shed corneas valuable information on the role of the molecules comes from a friction related disease, namely the lidwiper epiteliopathy (LWE). It was assumed that as LWE is associated with frictional and mechanical forces during blink, the increased friction between the lid wiper and ocular or contact lens surface and the reduced comfort might be related to insufficiency or altered composition of the ocular surface mucins [71]. The comfort levels and LWE were assessed in the right eyes of 50 experienced lens wearers (19 men, 31 women; mean age 32.1 ± 11.4 years; 31 asymptomatic and 19 symptomatic subjects classified by the Contact Lens Dry Eye Questionnaire). Significant correlation was revealed of LWE with decreased activity and quantity of MUC5AC, MUC4, and MUC16 [71].

Furthermore, it was reported that subclinical increase of corneal friction coefficient may trigger ocular inflammation thus providing an important link between the mechanistic and immunological component of dry eye [72].

### 3.3. On the Interplay of Secretory Mucins and MAM

Both mucins facilitate each other functions and the interplay between them and with other TF constituents is necessary for the proper functionality of the TF [1,2]. This was already discussed when highlighting the contribution of secretory mucins to the uniform spreading of TFLL and the mutually complimentary role of secretory mucins and MAM for the lubrication of the ocular surface. Such understanding is important as the commonly used model representation of layered TF frequently results in perception of the layers as separate entities while, in reality, they continuously synchronize and collaborate with each other.

Another example of interplay between secretory and MAMs comes from recent mathematical modeling work considering the shear thinning property of tears [9]. It turns out that, apart from MUC5AC, MAMs also play role here. MAMs are largely immobile, which results in viscosity gradient across the tear film with viscosity increasing from the air/tear surface towards the cornea. In dry eye conditions, MAMs in the glycocalyx become compromised and more of the mucin can diffuse into the AT. According to the model this results in reduction of the viscosity gradient in the TF which results in up to two times shorter BUT. Another potential reason for increased TF instability is the slip on the corneal surface, which may be exasperated by the loss of MAMs. Both mechanisms should be probed in controlled experiments monitoring the structural degradation of the corneal glycocalyx caused by DES [73]. The model predictions point to the important possibility to utilize the TF breakup patterns as a diagnostic tool for the assessment of the glycocalyx health and for ongoing ocular infections [54].

## 4. Conclusions

Important conclusion from the review of the interdisciplinary research accumulated up to now is that SM and MAM, which span across the entire tear fluid, from the corneal surface to the air/tear surface [1,2], may ensure the synchronization between the different layers of the TF. Indeed, secretory mucins may facilitate the spreading of TFLL [2,37,40] and the mucoaqueous gel ensures surface chemistry protection of the MAM at the surface of the corneal epithelium cells [11,14,17,26,27,28,29]. In turn the epithelium mucus ensures a viscosity gradient across the TF, which among others plays essential role for the shear thinning properties of human tears [9]. The latter property is essential for the tensile strength of the perched tear film at the interblink in open eye [11,17,19]. From other side the cooperation between SM and MAM ensures proper lubrication of the corneal surface, a property which if perturbed may trigger inflammation thus providing a link between the physical, mechanistic, and immunological component of DES [72]. The cooperation of SM and MAM between each other and with the rest of the TF constituents like TFLL opens novel and very important perspectives for the TF research. In a number of recent publications and reviews, we have highlighted the mechanisms of DES and among others the role of both secretory and membrane associated mucins to the performance of TF in health and disease [54,74,75]. Table 1 briefly summarizes the contributions of key mucins to the physical properties and stability of human tear film, the mucin alterations in dry eye pathologies, and possible mucin-oriented therapies of DES.

Table 1 also highlights number of novel mucin oriented therapeutic agents which emerged in the recent years. Diquafosol tetrasodium (3% ophthalmic solution, Diquas^®^; Santen, Osaka, Japan) is a dinucleotide compound and a purinergic P2Y_2_ receptor agonist that rapidly stimulates the secretion of tear fluid and SM and the expression of MAM. Detailed summary of the molecular scale mechanism is provided by Marcoulli et al. [76]. It has been shown to be effective in a broad range of DES phenotypes and especially in cases with decreased wettability of ocular surface, which are characterized typically with an instantaneous TF breakup at eye opening although patients display normal AT volume, no signs of MGD and mild to no ocular surface damage. In particular, 3% diquafosol sodium was able to gradually, after months of treatment, recover the short BUT pattern to normal (Figure 4) [63].

Rebamipide ophthalmic suspension (Mucosta^®^; Otsuka Pharmaceutical, Tokyo, Japan) is a mucin secretagogue which gradually enhances the production of mucin-like glycoproteins by human corneal epithelial cells and the expression of membrane-associated mucins MUC1, MUC4, and MUC16 [77,78]. Also, rebamipide is found to raise the number of goblet cells in the lid wiper and to facilitate the expression of cell surface proteins, epidermal growth factor receptor, MUC16 and MUC5AC [79]. These changes may alleviate the increased friction between the eyelid wiper and the cornea in dry eye [69]. Rebamipid may also beneficially influence the inflammation that takes place in dry eye [76]. Lacritin is a tear glycoprotein prosecretory mitogen that enhances basal tear secretion and epithelial homeostasis and currently undergoes a human clinical trial (NCT03226444) [76,80]. The topical application of 0.01% Lacripep^TM^ in mice DES model was found to result in 46% increase in tear secretion and reduction in lissamine green staining, and to recovery of the expression of corneal-specific cytokeratin K12, which is prevalent in the healthy ocular mucosal epithelium [76,80]. An emerging substance which has shown therapeutic promise when topically instilled in mice dry eyes is sulglycotide, a polysulfated glycopeptide derived from porcine duodenal mucin, which among others induced an increase in the numbers of goblet cells and enhanced the expression of MAM (MUC1, MUC4, and MUC16) and of the gel-forming mucin, MUC5AC [81].

The development of new drugs like diquafosol sodium, rebamipide, and lacritin—specifically targeting the mucus component of the TF—warrants gains of further understanding of the contribution of mucins to the functionality of the TF and to the better treatment of the diverse types of dry eye syndrome.

## Figures and Tables

**Table 1:** Summary of the contributions of key mucins, secretory gel forming and membrane associated, to the physical properties and stability of human tear film, the mucin alterations in dry eye pathologies, and possible mucin oriented therapies of DES. Detailed review of the biochemistry and immunology of the diverse mucin types present in ocular surface tissues and of the different DES subtypes can be found elsewhere [6,7,30,54,74,75].

Type of Mucins	Contribution to TF Physical Properties and Stability	Alterations in Pathology	Mucin Oriented Therapeutics
Secreted gel forming mucins: MUC5AC (most abundant), MUC5B, MUC2, and MUC19	The mucus gel ensures the shear thinning property of tears, facilitates the spreading of the lipid layer and acts as surface chemistry trap and barrier against invasion of lipids and exogenous agents towards the corneal surface.	Quantitative abnormalities of MUC5AC are observed in as diverse range of DES subtypes as aqueous tear deficient DE and evaporative DE	Diquafosol tetrasodium, lacritin
Membrane associated mucins: MUC1, MUC4, MUC16	MAM ensure ideal wettability and high lubricity of the ocular surface with MUC16 (having the longest hydrophilic ectodomain) considered especially critical. By ensuring no slip condition of the cornea and viscosity gradient across the TF, MAM might cooperate with secretory mucins on the shear thinning properties of tears.	Altered in decreased wettability DE subtype, characterized with normal AT volume and TFLL, but rapid breakup immediately at or <5 s after eye opening	Diquafosol tetrasodium, rebamipide

## Abbreviations

AT	Aqueous tear
BAM	Brewster angle microscopy
DES	Dry eye syndrome
HA	Hyaluronic acid
HCEC	Human corneal epithelium cells
MAM	Membrane associated mucins
MAG	Muco-aqueous gel
MGD	Meibomian gland dysfunction
NAC	N-acetylcysteine
NIBUT	Non-invasive breakup time
SM	Secretory mucins
TF	Tear film
TFLL	Tear film lipid layer
